# Risk factors, molecular features, and antibiotic susceptibility in carbapenem-resistant Enterobacterales infections among pediatric patients: a matched case-control study

**DOI:** 10.2478/abm-2026-0029

**Published:** 2026-08-31

**Authors:** Pintip Suchartlikitwong, Naris Kueakulpattana, Jiratchaya Sophonphan, Tanittha Chatsuwan

**Affiliations:** Department of Microbiology, Faculty of Medicine, Chulalongkorn University, Bangkok 10330, Thailand; Department of Pediatrics, Faculty of Medicine, Chulalongkorn University, Bangkok 10330, Thailand; Center of Excellence in Antimicrobial Resistance and Stewardship, Faculty of Medicine, Chulalongkorn University, Bangkok 10330, Thailand; Department of Statistics, the HIV Netherlands Australia Thailand Research Collaboration, Bangkok 10330, Thailand

**Keywords:** anti-bacterial agents, child, drug resistance, *Enterobacteriaceae*, microbial sensitivity tests, risk factors

## Abstract

**Background:**

Carbapenem-resistant Enterobacterales (CRE) infections are a growing global concern, with limited data in children.

**Objectives:**

This study aimed to identify risk factors for pediatric CRE infections and characterize their resistance genes and antibiotic susceptibility.

**Methods:**

From January 2020 to March 2024, pediatric patients diagnosed with CRE infections >48 h after admission at a tertiary-care hospital in Bangkok were consecutively enrolled. A matched case-control study was conducted, pairing each CRE case 1:1 with a carbapenem-susceptible Enterobacterales (CSE) control based on infection site and age. Risk factors were analyzed using conditional logistic regression. Carbapenemase genes were detected using multiplex polymerase chain reaction, and antibiotic susceptibilities were determined by minimal inhibitory concentration testing.

**Results:**

Seventy-six CRE cases and 76 matched CSE controls were included. Urinary tract infections were most common (42.1%). Independent risk factors included referral from other hospitals (adjusted odds ratios [aOR] 4.32; 95% CI 1.57–11.96), prior exposure to carbapenems (aOR 7.24; 95% CI 1.06–25.42), and third-generation cephalosporins (aOR 3.68; 95% CI 1.32–10.25). *Klebsiella pneumoniae* (72.4%) predominated among CRE isolates; *Escherichia coli* (52.6%) among CSE. Carbapenemase genes were detected in 76.3% of CRE isolates, mainly *bla*_NDM_ (60.3%) and *bla*_OXA-48_ (51.7%). Among non-carbapenems, amikacin showed the highest susceptibility against CSE (98.7%), and colistin against CRE isolates (88.2%). Ceftazidime/avibactam susceptibility was 100% among non-carbapenemase-producing CRE and 63.3% among CRE isolates with *bla*_OXA-48_.

**Conclusions:**

Pediatric CRE infections are associated with healthcare exposures and prior broad-spectrum antibiotic use. The high prevalence of carbapenemase genes and reduced antibiotic susceptibility emphasize the need for enhanced surveillance and targeted therapies.

Carbapenem-resistant *Enterobacterales* (CRE) are major causes of various healthcare-associated infections, including bloodstream, respiratory tract, intra-abdominal, and urinary tract infections (UTIs). The widespread prevalence of these critically drug-resistant bacteria poses a serious threat to public health due to limited therapeutic options, substantial economic burden, and increased mortality and morbidity rates. According to the World Health Organization's 2024 Bacterial Priority Pathogen List [1], CRE is ranked as one of the critical priority organisms, emphasizing the urgent need for new antibiotic development and effective infection control strategies. A global multicenter study conducted in 2019 across 64 medical centers worldwide reported an overall CRE rate of 4.5% among 8,787 Enterobacterales isolates, with regional rates as follows: Western Europe (1.2%), Eastern Europe (12.9%), the Asia-Pacific region (5.2%), and Latin America (5.8%) [2]. In Southeast Asia, a meta-analysis of extracted data from nations with ≥100 representative isolates obtained from 2010 onward estimated carbapenem resistance proportions among *Klebsiella* to be high (>5%) in Indonesia, the Philippines, Thailand and Vietnam, and among *E. coli* to be high (>5%) in Myanmar and Indonesia but low (<1%) in Cambodia, Malaysia, the Philippines, Singapore, Thailand and Vietnam [3]. The National Antimicrobial Resistance Surveillance in Thailand reported a rise in CRE prevalence from 0.7% to 13.2% in *K. pneumoniae* and from 0.5% to 3.9% in *E. coli* from 2010 to 2020 [4].

The main mechanism of resistance is the production of carbapenemase enzymes, classified as carbapenemase-producing Enterobacterales (CPE). Carbapenemases, a subgroup of beta-lactamases, hydrolyze penicillins, most cephalosporins, and to varying extents, carbapenems and monobactams. These enzymes are encoded by genes that are easily transferable and located on plasmid-associated transposable elements. Common carbapenemases include *K. pneumoniae* carbapenemase (KPC), New Delhi metallo-β-lactamase (NDM), Verona integron-encoded metallo-β-lactamase (VIM), imipenemase (IMP), and oxacillinase-48 (OXA-48) [5]. Additionally, carbapenem resistance can result from the combined effects of extended-spectrum beta-lactamase (ESBL) or AmpC enzyme production and reduced outer membrane permeability due to porin loss or efflux pump overexpression. These non-transferable resistant strains, classified as non-carbapenemase-producing CRE (non-CP-CRE), pose a lower risk of widespread dissemination [6]. A large-scale study conducted across 47 hospitals in Thailand revealed that the most common carbapenemase genes detected in clinical isolates were *bla*_NDM_, *bla*_OXA-48_, *bla*_VIM_, and *bla*_IMP_. These genes were identified in 74.9%, 22.4%, 4.6%, and 2.9% of carbapenem-resistant *E. coli* and in 52.9%, 54.1%, 2.8%, and 1.8% of carbapenem-resistant *K. pneumoniae* isolates, respectively [7].

A systematic review examining risk factors for CRE acquisition across all ages revealed that medical device use had the highest pooled estimate (odds ratio [OR] = 5.09; 95% CI, 3.38–7.67), followed by carbapenem use (OR = 4.71; 95% CI, 3.54–6.26) [8]. Risk factors for CRE infection in pediatric patients closely align with those in adults. Most cases occur in children with underlying comorbidities or a history of intensive care unit admission. Additional factors include prior or prolonged exposure to broad-spectrum antibiotics, use of medical devices, surgical interventions, and extended hospital stays [9,10,11].

The clinical and molecular epidemiology of CRE infections in pediatric patients remains limited in Thailand [5, 12]. This study primarily aimed to evaluate risk factors associated with CRE infections, in comparison to carbapenem-susceptible Enterobacterales (CSE) among children. The secondary objective was to investigate antimicrobial resistance genes and antibiotic susceptibility profiles in both CPE and non-CP-CRE isolates.

## Methods

### Study population and design

This retrospective study was conducted at a tertiary-care hospital in central Bangkok, Thailand, from January 2020 to March 2024. The hospital has a total of 1,500 beds, including 200 dedicated to pediatric care. This study included inpatients aged 0–18 yrs with CRE infections diagnosed more than 48 h after hospital admission. CRE was defined as an isolate intermediate or resistant to imipenem, meropenem, doripenem, or ertapenem according to the Clinical and Laboratory Standards Institute (CLSI) guidelines [13]. A total of 76 patients with CRE infections were consecutively included during the study period (**Figure 1**). The sample size was determined by including all eligible cases with available stored CRE isolates identified during the study period. Cases were identified from the hospital's infection control program database, which systematically tracks all CRE cases. Only the first isolates of CRE collected from multiple sites or on multiple dates in each patient were included in the analysis. Patients with Enterobacterales colonization, defined as the absence of clinical illness or corresponding treatment, were excluded.

**Figure 1.**
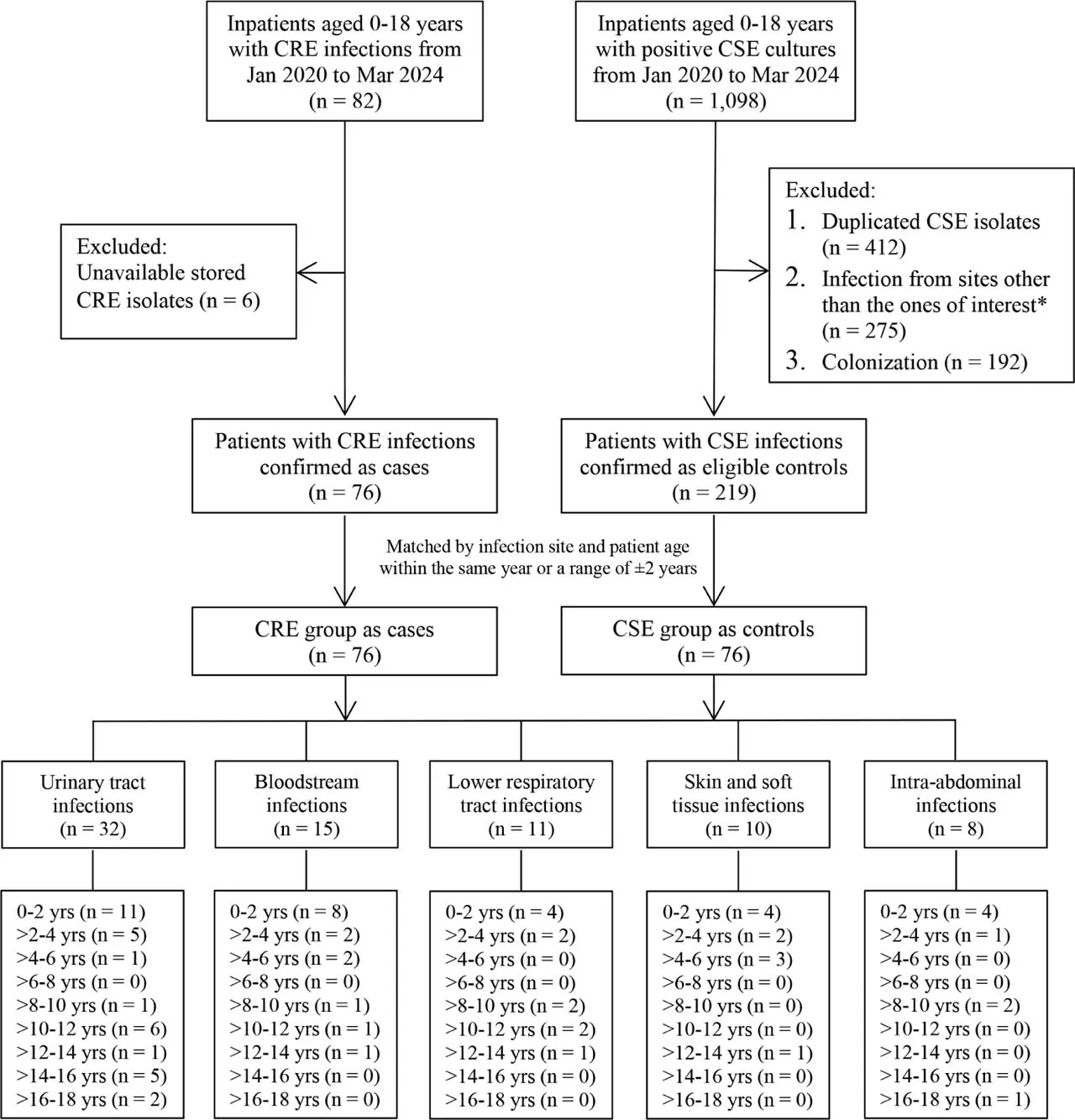
Flowchart of this matched case-control study. *Infection from sites other than the ones of interest includes upper respiratory tract specimens, cerebrospinal fluid, gastric content, bone marrow, catheter tips, conjunctival swab, pleural fluid, peritoneal dialysis fluid, breast milk, synovial fluid, and vaginal swab. CRE, carbapenem-resistant Enterobacterales; CSE, carbapenem-susceptible Enterobacterales; yrs, years.

A matched case-control study was conducted to evaluate the risk factors of CRE infections. For each patient with a CRE infection, a control patient was chosen from a pool of eligible inpatients with CSE infections diagnosed more than 48 h after hospital admission (**Figure 1**). The CSE group was selected to match the CRE group based on two criteria: (1) infection sites and (2) patient age within the same year or a range of ±2 yrs. If two or more CSE cases met the matching criteria for a given CRE case, a computer-generated random number was assigned to each eligible control, and the control with the lowest number was selected. Only the first isolates of CSE collected from multiple sites or on multiple dates in each patient were included in the analysis.

This study was reviewed and approved by the Institutional Review Board, Faculty of Medicine, Chulalongkorn University (IRB no. 752/65). Investigators ensured the anonymity of all patients.

### Data collection

Demographic data, underlying comorbidities, infection diagnosis, and microbiological results were extracted from the medical records. Clinical data of patients, including exposure to gram-negative-targeted antibiotic agents, a history of at least two hospitalizations, and undergoing major surgery within the past 6 months prior to a positive Enterobacterales culture, were reviewed. The presence of indwelling medical devices or catheters and mechanical ventilator use were assessed within 1 month prior to infection diagnosis.

Patients with Enterobacterales colonization—defined as the absence of compatible clinical signs or symptoms and no corresponding antimicrobial treatment—were excluded. Infections were diagnosed only when microbiologic evidence was accompanied by compatible clinical, laboratory, and/or radiologic findings, without an alternative source. The same criteria were applied to both CRE and CSE groups.

Regarding definitions of infection diagnosis, a central line-associated bloodstream infection (CLABSI) was diagnosed if at least one positive hemoculture was obtained from a patient with a central venous catheter in place and not related to another infection site. A UTI was defined as a positive urine culture with ≥10^4^ colony-forming units per mL of an organism in symptomatic patients. In patients with an indwelling urinary catheter in place for >2 d, the infection was subclassified as catheter-associated UTI (CAUTI). Skin and soft tissue infections were identified by Enterobacterales cultured from debrided tissue or drainage in patients with suppurative or inflammatory lesions. Lower respiratory tract infection included tracheobronchitis and pneumonia with clinical features (e.g., fever, leukocytosis, tachypnea, desaturation, increased secretions, and/or change in character of sputum). Ventilator-associated pneumonia (VAP) was defined as pneumonia with clinical and radiologic evidence in patients mechanically ventilated for more than 2 d, with the ventilator still in place on the event date. Intra-abdominal infection was defined as originating from the gastrointestinal or hepatobiliary tract, with or without extension into the peritoneal space or other sterile regions of the abdominal cavity. All cases were reviewed by the study investigator, an infectious diseases specialist, to ensure uniform application of the criteria and consistent differentiation between infection and colonization.

### Microbial identification and susceptibility testing

All isolates were identified using matrix-assisted laser desorption ionization time-of-flight mass spectrometry (bioMérieux, Marcy-l'Étoile, France). The antibiotic susceptibility patterns of CRE and CSE isolates were assessed using the VITEK 2XL automated system (bioMérieux, Marcy-l'Étoile, France) with VITEK 2 AST-N416 cards. The minimal inhibitory concentrations (MICs) of CRE isolates were determined using the agar dilution method for all antibiotics, except piperacillin/tazobactam and ceftazidime/avibactam, which were assessed using Etest strips (bioMérieux, Marcy-l'Étoile, France). Colistin, tigecycline, and fosfomycin MICs were evaluated using the broth microdilution method. Antibiotic susceptibility results were interpreted according to the CLSI guidelines, except for tigecycline, which followed the European Committee on Antimicrobial Susceptibility Testing guidelines [14].

### Detection of resistance genes in CRE isolates

DNA extraction for each CRE isolate was performed using the boiling method. Five colonies of each isolate were suspended in 200 μL of sterile distilled water and boiled for 10 min. The boiled suspension was then centrifuged at 13,000 rpm at room temperature for 10 min. After removing cell debris, the supernatant was used as template DNA and stored at −20°C for polymerase chain reaction (PCR) experiments. Using previously described primers [15,16,17,18,19,20], (ESBL; *bla*_SHV_, *bla*_TEM_, *bla*_OXA-1_, *bla*_CTX-M_, and *bla*_VEB_) (**Supplementary Table 1**) and carbapenemase genes (*bla*_IMP_, *bla*_VIM_, *bla*_OXA-48_, *bla*_NDM_, and *bla*_KPC_) (**Supplementary Table 2**) were detected by multiplex PCR. Non-carbapenemase-producing CRE isolates were confirmed by negative results from the modified carbapenem inactivation method.

### Statistical analysis

Continuous data were reported as medians with interquartile ranges (IQR), while categorical data were presented as percentages. Differences in continuous data between two groups were analyzed using the Wilcoxon rank-sum test, whereas differences in proportions between two groups were assessed using the Chi-square test or Fisher's exact test. Variables associated with CRE infection were evaluated using conditional logistic regression. Multivariable models were developed by adjusting for covariates with a *P*-value <0.1 in the univariable analysis, using stepwise backward likelihood ratio selection to determine the final model. All reported *P*-values were two-sided, with statistical significance defined as *P* <0.05. Statistical analyzes were performed using Stata version 18.0 (StataCorp LLC., College Station, Texas). MIC50 and MIC90 represent the MIC required to inhibit the growth of 50% and 90% of all CRE isolates, respectively.

## Results

### Baseline characteristics of CRE and CSE patients

Over a 4-year period, a total of 76 cases with CRE infections were included in this study. An equal number of control patients with CSE infections were matched to the CRE cases by site of infection and age. The sites of Enterobacterales infection included UTIs (n = 32, 42.1%), bloodstream infections (n = 15, 19.7%), lower respiratory tract infections (n = 11, 14.5%), skin and soft tissue infections (n = 10, 13.2%), and intra-abdominal infections (n = 8, 10.5%). Among UTI cases, 4 of 32 patients with CRE (12.5%) had CAUTI, while none in the CSE did. The majority of bloodstream infections were CLABSI, identified in 13 of 15 (86.7%) CRE cases and 9 of 15 (60.0%) CSE cases. Among lower respiratory tract infections, VAP was the most common in both groups, occurring in 10 of 11 (90.9%) cases in each group. The distribution of patients by age strata and infection site is shown in **Figure 1**. The median age was 3.1 yrs (IQR, 0.9–10.6) in the CRE group and 3.5 yrs (IQR, 0.8–10.8) in the CSE group. Gender distribution was comparable between groups, with males comprising 52.6% of the CRE group and 56.6% of the CSE group. Notably, CRE patients had a significantly longer median hospital stay compared with CSE patients (65 d [IQR 24–119] vs. 18 d [IQR 7–42]; *P* < 0.001).

### Risk factors for CRE infections

Compared to patients with CSE infections, those with CRE infections were significantly more likely to be referred from other primary or secondary care hospitals (OR = 4.14; 95% CI, 1.81–9.45; *P* = 0.001) and to require admission to the intensive care unit (OR = 2.86; 95% CI, 1.21–6.75; *P* = 0.017). Underlying hepatobiliary or gastrointestinal diseases were also more common among CRE patients (OR = 3; 95% CI, 1.09–8.25; *P* = 0.03). Prior exposure to at least one gram-negative-targeted antibiotic within 6 months was more frequent in the CRE group. This included carbapenems (OR = 11; 95% CI, 3.37–35.87; *P* < 0.001), older β-lactam/β-lactamase inhibitors (OR = 3.86; 95% CI, 1.68–8.86; *P* < 0.001), third-generation cephalosporins (OR = 4.29; 95% CI, 1.88–9.76; *P* < 0.001), fluoroquinolones (OR = 4; 95% CI, 1.5–10.66; *P* = 0.01), and aminoglycosides (OR = 3; 95% CI, 1.19–7.56; *P* = 0.02). Additional factors associated with CRE infection included multiple prior hospitalizations (OR = 3.25; 95% CI, 1.47–7.18; *P* < 0.001), presence of a central line (OR = 4.33; 95% CI, 1.78–10.53; *P* < 0.001), use of a nasogastric or orogastric tube (OR = 2.7; 95% CI, 1.31–5.58; *P* = 0.01), and mechanical ventilation (OR = 2.83; 95% CI, 1.12–7.19; *P* = 0.03).

On multivariable analysis, independent risk factors for CRE infection included referral from other hospitals (aOR = 4.32; 95% CI, 1.57–11.96; *P* = 0.005), prior exposure to carbapenems (aOR = 7.24; 95% CI, 1.06–25.42; *P* = 0.002), and third-generation cephalosporins (aOR = 3.68; 95% CI, 1.32–10.25; *P* = 0.013) (**Table 1**).

**Table 1.** Univariate and multivariate analysis to evaluate risk factors associated with CRE infections

**Variables**	**CRE patients (n = 76)**	**CSE patients (n = 76)**	**Univariate analysis**		**Multivariate analysis**	

			**OR (95% CI)**	***P***	**aOR (95% CI)**	***P***
Referral from other hospitals	44 (57.9%)	22 (28.9%)	4.14 (1.81–9.45)	0.001	4.32 (1.57–11.96)	0.005
Intensive care unit	36 (47.4%)	23 (30.3%)	2.86 (1.21–6.75)	0.017		
**Comorbidities**						
Malignancies	13 (17.1%)	14 (18.4%)	0.86 (0.29–2.55)	0.78		
Genitourinary diseases	11 (14.5%)	17 (22.4%)	0.4 (0.13–1.28)	0.12		
Hepatobiliary or gastrointestinal diseases	21 (27.6%)	11 (14.5%)	3.0 (1.09–8.25)	0.03		
Cardiovascular or respiratory diseases	17 (22.4%)	11 (14.5%)	2.5 (0.78–7.97)	0.12		
Neurological or cutaneous diseases	13 (17.1%)	8 (10.5%)	1.83 (0.66–4.96)	0.23		
**Antibiotic exposure in the past 6 months**						
• Carbapenems	47 (61.8%)	16 (21.1%)	11.0 (3.37–35.87)	<0.001	7.24 (1.06–25.42)	0.002
• Older β-lactam/β-lactamase inhibitors¶	48 (63.2%)	28 (36.8%)	3.86 (1.68–8.86)	<0.001		
• 3^rd^ generation cephalosporins	47 (61.8%)	23 (30.3%)	4.29 (1.88–9.76)	<0.001	3.68 (1.32–10.25)	0.013
• Fluoroquinolones	26 (34.2%)	11 (14.5%)	4.0 (1.5–10.66)	0.01		
• Aminoglycosides	23 (30.3%)	11 (14.5%)	3.0 (1.19–7.56)	0.02		
**Multiple hospitalizations**	37 (48.7%)	18 (23.7%)	3.25 (1.47–7.18)	<0.001		
**Undergoing major surgery**	37 (48.7%)	26 (34.2%)	1.71 (0.89–3.31)	0.11		
**Indwelling medical devices**						
• Central line	44 (57.9%)	24 (31.6%)	4.33 (1.78–10.53)	<0.001		
• Arterial line	17 (22.4%)	11 (14.5%)	2.0 (0.75–5.33)	0.17		
• Nasogastric or orogastric tube	42 (55.3%)	24 (31.6%)	2.7 (1.31–5.58)	0.01		
• Abdominal drainage	21 (27.6%)	11 (14.5%)	2.11 (0.96–4.67)	0.06		
**Mechanical ventilation**	27 (35.5%)	16 (21.1%)	2.83 (1.12–7.19)	0.03		

CRE, carbapenem-resistant Enterobacterales.

### Isolation of Enterobacterales

In this study, CRE was isolated from 76 patients, with *K. pneumoniae* (n = 55, 72.4%) as the most common isolate, followed by *E. coli* (n = 16, 21.0%) and *E. cloacae* complex (n = 5, 6.6%). Among the 76 CSE cases, isolates included *E. coli* (n = 40, 52.6%) as the most common, followed by *K. pneumoniae* (n = 25, 32.9%) and *E. cloacae* complex (n = 11, 14.5%).

### Antimicrobial resistance genes

Among the 76 CRE isolates, carbapenemase genes were detected in 58 isolates (76.3%), designated as CPE (**Table 2**). Carbapenemase genes were not detected in the remaining CRE isolates, which were classified as non-CP-CRE, including 11 *K. pneumoniae*, 2 *E. coli*, and all 5 *E. cloacae* complex isolates. Among the CPE isolates, the carbapenemase genes *bla*_NDM_ (n = 35, 60.3%) and *bla*_OXA-48_ (n = 30, 51.7%) were the only ones detected. Specifically, *bla*_NDM_ was identified in 24 *K. pneumoniae* and 11 *E. coli* isolates, while *bla*_OXA-48_ was present in 24 *K. pneumoniae* and 6 *E. coli* isolates. No *bla*_IMP_, *bla*_VIM_, or *bla*_KPC_ genes were detected. Seven CPE isolates (12.1%) harbored both *bla*_NDM_ and *bla*_OXA-48_, comprising 4 *K. pneumoniae* and 3 *E. coli* isolates. All CRE isolates carried at least one ESBL gene, with 24% harboring one, 36% two, 29% three, and 12% four ESBL genes. Co-existence of 2 or more ESBL types was detected in 13 (81%) *E. coli*, 44 (80%) *K. pneumoniae,* and 1 (20%) *E. cloacae* complex isolate. The most prevalent ESBL gene was *bla*_CTX-M_ (n = 67, 88.2%), followed by *bla*_TEM_ (n = 49, 64.5%), *bla*_SHV_ (n = 35, 46.1%), and *bla*_OXA-1_ (n = 23, 30.3%). No *bla*_VEB_ genes were detected in these isolates.

**Table 2.** Prevalence of ESBL and carbapenemase genes among 76 CRE isolates

**Genes**	**CPE (n = 58)**	**Non-CP-CRE (n = 18)**	**Total (n = 76)**
**Extended-spectrum beta-lactamase**			
*bla*_SHV_	27 (46.6%)	8 (44.4%)	35 (46.1%)
*bla*_TEM_	38 (65.5%)	11 (61.1%)	49 (64.5%)
*bla*_OXA-1_	17 (29.3%)	6 (33.3%)	23 (30.3%)
*bla*_CTX-M_	53 (91.4%)	14 (77.8%)	67 (88.2%)
*bla*_VEB_	0 (0.0%)	0 (0.0%)	0 (0.0%)
**Carbapenemase**			
*bla*_NDM_	35 (60.3%)	-	35 (46.1%)
*bla*_OXA-48_	30 (51.7%)	-	30 (39.5%)
*bla*_NDM_ + *bla*_OXA-48_	7 (12.1%)	-	7 (9.2%)
*bla*_IMP_	0 (0.0%)	-	0 (0.0%)
*bla*_VIM_	0 (0.0%)	-	0 (0.0%)
*bla*_KPC_	0 (0.0%)	-	0 (0.0%)

CRE, carbapenem-resistant Enterobacterales; ESBL, extended-spectrum beta-lactamase.

### Antibiotic susceptibility profiles

Overall, CRE isolates showed lower susceptibility to all antibiotics compared to CSE isolates (**Table 3**). Among non-carbapenems, CSE isolates were most susceptible to amikacin (98.7%), followed by piperacillin/tazobactam (86.8%), cefepime (76.3%), gentamicin (75.0%), ceftriaxone (63.2%), and amoxicillin/clavulanate (60.5%). In contrast, CRE isolates showed the highest susceptibility to colistin (88.2%), followed by amikacin (69.7%), gentamicin (50.0%), and ceftazidime/avibactam (50.0%).

**Table 3.** Antibiotic susceptibility profiles of CRE and CSE isolates

**Antibiotic agents**	**Isolates susceptible, n (%)**	
	**CRE isolates (n = 76)**	**CSE isolates (n = 76)**
Amikacin (AMK)	53 (69.7%)	75 (98.7%)
Gentamicin (GEN)	38 (50.0%)	57 (75.0%)
Ciprofloxacin (CIP)	4 (5.3%)	32 (42.1%)
Ceftriaxone (CRO)	2 (2.6%)	48 (63.2%)
Cefepime (FEP)	6 (7.9%)	58 (76.3%)
Imipenem (IPM)	8 (10.5%)	76 (100.0%)
Meropenem (MEM)	17 (22.4%)	76 (100.0%)
Ertapenem (ETP)	3 (3.9%)	76 (100.0%)
Trimethoprim/sulfamethoxazole (SXT)	15 (19.7%)	35 (46.1%)
Piperacillin/tazobactam (TZP)	1 (1.3%)	66 (86.8%)
Amoxicillin/clavulanate (AMC)	0 (0.0%)	46 (60.5%)

CRE, carbapenem-resistant Enterobacterales; CSE, carbapenem-susceptible Enterobacterales.

**Table 4** summarizes the susceptibility rates, and **Table 5** presents the MIC50, MIC90, and MIC ranges for selected antibiotics in CRE isolates, categorized as CPE based on carbapenemase genes and non-CP-CRE. Non-CP-CPE isolates were 100% susceptible to ceftazidime/avibactam, while only *bla*_OXA-48_-positive CPE isolates showed 63.3% susceptibility. Non-CP-CPE isolates also demonstrated higher susceptibility to amikacin (88.9% vs. 63.7%, *P* = 0.043), meropenem (72.2% vs. 6.9%, *P* < 0.001), and imipenem (44.4% vs. 0.0%, *P* < 0.001). Conversely, CPE isolates were more susceptible to colistin (91.4% vs. 77.8%, *P* = 0.119) and fosfomycin (36.2% vs. 11.1%, *P* = 0.043). Susceptibility to tigecycline was low in both groups (13.8% in CPE vs. 11.1% in non-CP-CRE).

**Table 4.** Susceptibility rates for selected antibiotics in CRE isolates, categorized into CPE based on carbapenemase genes and non-CP-CRE

**Antibiotic agents**	***bla*_NDM_ (n = 35)**	***bla*_OXA-48_ (n = 30)**	***bla*_NDM_ + *bla*_OXA-48_ (n = 7)**	**Total CPE (n = 58)**	**Non-CP-CRE (n = 18)**	***P***
AMK	26 (74.3%)	16 (53.3%)	5 (71.4%)	37 (63.7%)	16 (88.9%)	0.043
GEN	18 (51.4%)	12 (40.0%)	2 (28.6%)	28 (48.3%)	10 (55.6%)	0.589
IPM	0 (0.0%)	0 (0.0%)	0 (0.0%)	0 (0.0%)	8 (44.4%)	<0.001
MEM	0 (0.0%)	4 (13.3%)	0 (0.0%)	4 (6.9%)	13 (72.2%)	<0.001
ETP	0 (0.0%)	0 (0.0%)	0 (0.0%)	0 (0.0%)	3 (16.7%)	0.002
CZA	1 (2.9%)	19 (63.3%)	0 (0.0%)	20 (34.5%)	18 (100.0%)	<0.001
CST^#^	31 (88.6%)	27 (90.0%)	5 (71.4%)	53 (91.4%)	14 (77.8%)	0.119
TGC	5 (14.3%)	4 (13.3%)	1 (14.3%)	8 (13.8%)	2 (11.1%)	0.769
FOF	15 (42.9%)	9 (30.0%)	3 (42.9%)	21 (36.2%)	2 (11.1%)	0.043

AMK, amikacin; GEN, gentamicin; IPM, imipenem; MEM, meropenem; ETP, ertapenem; CZA, ceftazidime/avibactam; CST, colistin; TGC, tigecycline; FOF, fosfomycin.

# Using the intermediate breakpoint following the Clinical and Laboratory Standards Institute (CLSI) 2024.

**Table 5.** Minimal inhibitory concentrations for selected antibiotics in CRE isolates, categorized as CPE based on carbapenemase genes and non-CP-CRE.

	***bla*_NDM_ (n = 35)**			***bla*_OXA-48_ (n = 30)**			***bla*_NDM_ + *bla*_OXA-48_ (n = 7)**			**Non-CP-CRE (n =1 8)**		
			
	**MIC50**	**MIC90**	**MIC range**	**MIC50**	**MIC90**	**MIC range**	**MIC50**	**MIC90**	**MIC range**	**MIC50**	**MIC90**	**MIC range**
AMK	4	128	1–>512	4	>512	0.25–>512	4	64	1–128	2	4	0.5–512
GEN	2	256	0.5–>512	32	>512	0.25–>512	32	128	2–512	1	128	0.125–512
IPM	128	256	4–512	16	256	2–512	128	256	64–512	2	4	0.06–64
MEM	128	128	8–256	32	128	0.5–256	128	128	128–128	1	4	0.06–64
ETP	256	512	4–>512	256	512	2–>512	512	512	256–512	8	256	0.5–512
CZA	>256/4	>256/4	1/4–>256/4	1.5/4	>256/4	0.125/4–>256/4	>256/4	>256/4	>256/4–>256/4	1/4	2/4	0.19/4–2/4
CST^#^	1	8	0.125–16	1	2	0125–32	1	8	1–16	0.5	8	0.25–16
TGC	2	4	0.25–8	4	8	0.5–8	2	4	0.5–8	2	4	0.125–4
FOF	128	>512	1–>512	512	>512	2–>512	>512	>512	4–>512	256	>512	2–>512

AMK, amikacin; GEN, gentamicin; IPM, imipenem; MEM, meropenem; ETP, ertapenem; CZA, ceftazidime/avibactam; CST, colistin; TGC, tigecycline; FOF, fosfomycin.

# Using the intermediate breakpoint following the Clinical and Laboratory Standards Institute (CLSI) 2024.

**Supplementary Table 3** shows the susceptibility rates, MIC50, MIC90, and MIC ranges for selected antibiotics in CRE isolates, grouped by their species. *E. coli* had the highest susceptibility to colistin (100%), tigecycline (43.8%), and fosfomycin (68.8%), compared to *K. pneumoniae* (85.8%, 5.5%, and 21.8%) and *E. cloacae* complex (80.0%, 0.0%, and 0.0%).

## Discussion

This study evaluated risk factors associated with CRE infections compared to CSE among pediatric patients over a 4-year period. In addition, we characterized carbapenemase and ESBL genes in CRE isolates, and performed a comprehensive analysis of antibiotic susceptibility profiles, including MIC data. To our knowledge, this is the first study to investigate the clinical and molecular epidemiology of CRE infections in Thai children.

In this study, referral from other healthcare facilities and prior exposure to carbapenems and third-generation cephalosporins emerged as independent risk factors for CRE infection among pediatric patients. These findings are consistent with previous studies, highlighting the role of healthcare-associated exposures and prior antibiotic use in the selection and transmission of multidrug-resistant organisms [10, 11, 21,22,23,24,25]. Referral from other hospitals likely reflects increased healthcare contact and exposure to environments with a higher prevalence of CRE, contributing to colonization. This trend is consistent with the National Antimicrobial Resistance Surveillance in Thailand report, which highlights the growing threat of CRE across hospitals nationwide. The increased risk associated with prior antibiotic exposure is likely due to disruptions in gut microbiota caused by broad-spectrum agents, which promote the selection of resistant bacteria. A large nationwide study in Singapore reported that prior carbapenem exposure was 3 times more strongly associated with non-CP-CRE acquisition than with CPE, underscoring the role of antibiotic selection pressure and the importance of antimicrobial stewardship [23, 26]. In our cohort, 23.7% of CRE cases were non-CP-CRE, while the majority harbored carbapenemase genes. Unlike non-CP-CRE, which often emerges in a patient's own microbiota under selective pressure from prior antibiotic exposure (endogenous selection), CPE is predominantly acquired via clonal spread or horizontal gene transfer [27, 28]. This suggests that inadequate infection control practices—such as poor hand hygiene, insufficient isolation precautions, and suboptimal environmental cleaning—may contribute to CPE dissemination in our setting.

Among the CRE isolates in our study, CPE were found in a higher proportion (76.3%), with *K. pneumoniae* being the most prevalent CPE organism, followed by *E. coli*. *bla*_NDM_ (60.3%) and *bla*_OXA-48_ (51.7%) were identified as the two most common carbapenemase genes in this study. These results are consistent with previous studies in Thailand [7, 29, 30]. However, the distribution of carbapenemase genes in Enterobacterales varies by country and region [31]. A recent global surveillance study showed that, among meropenem-non-susceptible Enterobacterales, *bla*_KPC_ was the most frequent carbapenemase gene in North America and Europe, while *bla*_NDM_ was the most prevalent in the Asia Pacific, Latin America, and the Middle East-Africa regions [32]. ESBL genes were also detected in all of our CRE isolates. The co-existence of ESBL and carbapenemase genes is increasingly common among Enterobacterales in Thailand, which acquire both genes via plasmid-mediated mechanisms and may also harbor genes encoding resistance to other drug classes [7, 33, 34]. In our study, *bla*_CTX-M_ was identified as the most predominant ESBL gene (88.2%). This finding aligns with a global study reporting that, among all ESBL-producing *E. coli* and *K. pneumoniae* isolates, *bla*_CTX-M_ constituted the majority (87.4% and 86.7%, respectively), while other genotypes, including *bla*_VEB_, *bla*_TEM_, *bla*_SHV_, *bla*_GES_, and *bla*_PER_, were detected at lower frequencies [35].

All CRE isolates in our study exhibited high resistance to multiple antibiotic classes, including carbapenems, cephalosporins, older β-lactam/β-lactamase inhibitors, and fluoroquinolones. In our setting, *bla*_NDM_ and *bla*_OXA-48_ were the two most common carbapenemase genes detected among CPE isolates. For such cases, newer β-lactam/β-lactamase inhibitors, such as ceftazidime/avibactam combined with aztreonam, appear to be the most effective treatment option [36, 37]. Aztreonam provides inhibitory activity against isolates harboring *bla*_NDM_, while ceftazidime/avibactam is effective against those harboring *bla*_OXA-48_. Cefiderocol monotherapy is also a preferred treatment for isolates carrying *bla*_NDM_ and serves as an alternative for those with *bla*_OXA-48_, as recommended by the Infectious Diseases Society of America (IDSA) [37]. However, neither aztreonam nor cefiderocol is currently available for pediatric patients in Thailand, leaving ceftazidime/avibactam as the only viable option for CRE isolates with *bla*_OXA-48_. Notably, 63.3% of these isolates were susceptible to ceftazidime/avibactam in our study. For non-CP-CRE isolates, we observed significantly higher susceptibilities to meropenem (72.2%) and cef-tazidime/avibactam (100%) compared with CPE isolates. This supports the use of extended-infusion meropenem or newer β-lactam/β-lactamase inhibitors, such as ceftazidime/avibactam, for treating infections caused by non-CP-CRE outside the urinary tract, in accordance with IDSA recommendations [37].

Given these findings, understanding the type of carbapenemase produced by CRE isolates is crucial for guiding treatment decisions, as specific newer β-lactam antibiotics target specific carbapenemases. However, the availability of effective antibiotics against CRE is limited, and testing for carbapenemase production, whether through nucleic acid or antigen testing, is not routinely performed in Thailand's clinical laboratories. Due to the high susceptibility of our CRE isolates to colistin (88.2%) and amikacin (69.7%), combination antibiotic therapy with one or both active second-line agents was recommended. This included colistin or amikacin combined with other active agents, such as fluoroquinolones, tigecycline, fosfomycin, or high-dose extended-infusion meropenem (for isolates with a meropenem MIC ≤8 μg/mL) [4, 38]. These combinations were tailored to address the limited availability of newer agents and the resistance profiles observed in our study. Nevertheless, the use of fluoroquinolones in pediatric patients should be approached with caution due to the risk of musculoskeletal adverse events, such as tendonitis and tendon rupture [39].

This study has some limitations. First, as a retrospective single-center case-control study, the findings may not be generalizable, and the study design, rather than a cohort approach, limits causal interpretation of the results. Second, due to the retrospective nature of the study, some risk factor variables, such as non-invasive medical devices, over-the-counter antibiotics, and treatments received at other hospitals, may have been incompletely captured if not documented in the medical records. In addition, duration and dosage of prior antibiotic exposure were not verified through pharmacy records, which may have included short courses such as exposures of less than 48 h. Third, we could not match cases and controls by time period due to the limited number of eligible controls, which may have introduced temporal confounding related to changes in infection epidemiology over the 4-year study period. Finally, the absence of molecular epidemiological investigations, such as multilocus sequence typing, limited our ability to assess the clonal spread and diversity of CRE isolates or predict the likelihood of outbreaks within the hospital.

## Conclusions

Referral from other hospitals and prior exposure to carbapenems and third-generation cephalosporins were independent risk factors for CRE infection. *K. pneumoniae* was the most common CRE isolate, with *bla*_NDM_ and *bla*_OXA-48_ as the predominant carbapenemase genes. High susceptibility rates to ceftazidime/avibactam, amikacin, and colistin suggest these agents as potential treatments. These findings provide valuable insights into the clinical epidemiology, antibiotic resistance genes, and susceptibility profiles of CRE infections in pediatric patients, highlighting the need for tailored therapeutic strategies and strengthened infection control measures to combat antimicrobial resistance.

## Figures and Tables

**Table S1.** Primers for detection of extended-spectrum beta-lactamase genes

**Genes**	**Primer name**	**Sequence (5′-3′)**	**Product size (bp)**
*bla*_SHV-like_	SHV-F	AGGATTGACTGCCTTTTTG	392
	SHV-R	ATTTGCTGATTTCGCTCG	
*bla*_TEM-like_	TEM-C	ATCAGCAATAAACCAGC	516
	TEM-H	CCCCGAAGAACGTTTTC	
*bla*_OXA-1-like_	OXA-F	ATATCTCTACTGTTGCATCTCC	619
	OXA-R	AAACCCTTCAAACCATCC	
*bla*_CTX-M-like_	CTXM-A	CGCTTTGCGATGTGCAG	550
	CTXM-B	ACCGCGATATCGTTGGT	
*bla*_VEB-like_	VEB-A	CCTTTTGCCTAAAACGTGGA	216
	VEB-B	TGCATTTGTTCTTCGTTTGC	

bp, base pairs.

**Table S2.** Primers for detection of carbapenemase genes

**Genes**	**Primer name**	**Sequence (5′-3′)**	**Product size (bp)**
*bla*_IMP-like_	IMP-F	GGAATAGAGTGGCTTAAYTCT	188
	IMP-R	CCAAACYACTASGTTATCT	
*bla*_VIM-like_	VIM-F	GATGGTGTTTGGTCGCATA	390
	VIM-R	CGAATGCGCAGCACCAG	
*bla*_OXA-48-like_	OXA-48-F	GCGTGGTTAAGGATGAACAC	438
	OXA-48-R	CATCAAGTTCAACCCAACCG	
*bla*_NDM-like_	NDM-F	GGTTTGGCGATCTGGTTTTC	621
	NDM-R	CGGAATGGCTCATCACGATC	
*bla*_KPC-like_	KPC-F	CGTCTAGTTCTGCTGTCTTG	798
	KPC-R	CTTGTCATCCTTGTTAGGCG	

bp, base pairs.

**Table S3.** Susceptibility rates and minimal inhibitory concentrations for studied antibiotics in CRE isolates, grouped by their species

**Types of CRE**	**Total isolates**	**AMK**	**GEN**	**CIP**	**CRO**	**FEP**	**IPM**	**MEM**	**ETP**	**SXT**	**TZP**	**CZA**	**CST^#^**	**TGC**	**FOF**
***Klebsiella pneumoniae***	55	35 (63.6%)	31 (56.4%)	3 (5.5%)	1 (1.8%)	4 (7.3%)	4 (7.3%)	12 (21.8%)	2 (3.6%)	11 (20.0%)	1 (1.8%)	29 (52.7%)	47 (85.8%)	3 (5.5%)	12 (21.8%)
	MIC50	4	2	128	>512	512	64	32	256	64/1216	>256/4	2/4	1	2	256
	MIC90	>512	>512	>512	>512	>512	256	128	512	>512/9728	>256/4	>256/4	8	8	>512
	MIC range	0.25–>512	0.125–>512	0.125–>512	1–>512	0.125–>512	0.06–512	0.06–256	0.5–>512	0.25/4.75–>512/9728	4/4–>256/4	0.125/4–>256/4	0.125–32	0.125–8	16–>512

***Escherichia coli***	16	13 (81.3%)	5 (31.3%)	0 (0.0%)	1 (6.3%)	1 (6.3%)	1 (6.3%)	1 (6.3%)	0 (0.0%)	2 (12.5%)	0 (0.0%)	4 (25.0%)	16 (100%)	7 (43.8%)	11 (68.8%)
	MIC50	4	32	256	>512	512	64	64	256	128/2432	>256/4	>256/4	0.5	1	8
	MIC90	64	128	>512	>512	>512	256	128	512	256/4864	>256/4	>256/4	1	1	>512
	MIC range	1–512	1–512	0.5–>512	1–>512	2–>512	0.5–256	0.5–128	4–512	0.5/9.5–>512/9728	32/4–>256/4	0.75/4–>256/4	0.125–2	0.25–2	1–>512

***Enterobacter cloacae* complex**	5	5 (100%)	2 (40.0%)	1 (20.0%)	0 (0.0%)	1 (20.0%)	3 (60.0%)	4 (80.0%)	1 (20.0%)	2 (40.0%)	0 (0.0%)	5 (100%)	4 (80.0%)	0 (0.0%)	0 (0.0%)
	MIC50	4	8	16	256	4	0.5	1	8	32/608	32/4	0.75/4	0.5	1	256
	MIC90	4	32	>512	512	512	4	4	256	256/4864	256/4	2/4	16	2	512
	MIC range	0.5–4	0.125–32	0.03–>512	64–>512	1–>512	0.25–4	0.125–4	0.5–256	0.25/4.75–256/4864	32/4–256/4	0.25/4–2/4	0.25–16	1–2	128–>512

**Total CRE isolates**	76	53 (69.7%)	38 (50.0%)	4 (5.3%)	2 (2.6%)	6 (7.9%)	8 (10.5%)	17 (22.4%)	3 (3.9%)	15 (19.7%)	1 (1.3%)	38 (50.0%)	67 (88.2%)	10 (13.2%)	23 (30.3%)
	MIC50	4	8	128	>512	512	32	32	256	64/1216	>256/4	>256/4	1	2	256
	MIC90	512	512	>512	>512	>512	256	128	512	>512/9728	>256/4	>256/4	8	4	512
	MIC range	0.25–>512	0.125–>512	0.03–>512	1–>512	0.125–>512	0.06–512	0.06–256	0.5–>512	0.25/4.75–>512/9728	4/4–>256/4	0.125/4–>256/4	0.125–32	0.125–8	1–>512

AMK, amikacin; GEN, gentamicin; CIP, ciprofloxacin; CRO, ceftriaxone; FEP, cefepime; IPM, imipenem; MEM, meropenem; ETP, ertapenem; SXT, trimethoprim/sulfamethoxazole; TZP, piperacillin/tazobactam; CZA, ceftazidime/avibactam; CST, colistin; TGC, tigecycline; FOF, fosfomycin.

# Using the intermediate breakpoint following the Clinical and Laboratory Standards Institute (CLSI) 2024.

## Abbreviations

aOR: adjusted odds ratios
CAUTI: catheter-associated UTI
CLABSI: central line-associated bloodstream infection
CLSI: clinical and laboratory standards institute
CPE: carbapenemase-producing *Enterobacterales*
CRE: carbapenem-resistant *Enterobacterales*
CSE: carbapenem-susceptible *Enterobacterales*
ESBL: extended-spectrum beta-lactamase
IQR: interquartile ranges, MICs, minimal inhibitory concentrations
Non-CP-CRE: non-carbapenemase-producing carbapenem-resistant *Enterobacterales*
OR: odds ratios
PCR: polymerase chain reaction
UTI: urinary tract infection
VAP: ventilator-associated pneumonia
Yrs: years
